# Supplementary material for: Intra- and interspecific diversity in a tropical plant clade alter herbivory and ecosystem resilience
Source: eLife. 2024 Apr 25;12:RP86988. doi: 10.7554/eLife.86988 (PMC11045218; doi:10.7554/eLife.86988)
Supplement: Supplementary file 1. [file elife-86988-supp1.docx]

**Supplementary file 1.** Mean parameter estimates and probability of direction (PD) for the effects of increases in intraspecific diversity, interspecific richness, water availability and insect richness on measures of herbivory, plant mortality, and insect richness

| Site | Predictor variable | Response variable | Mean parameter estimate | PD |
| --- | --- | --- | --- | --- |
| All | Intraspecific richness | Percent herbivory | -0.87 | 63.59% |
| All | Intraspecific richness | Presence of damage | -3.26 | 71.60% |
| All | Intraspecific richness | Variance in herbivory | -9.97 | 57.31% |
| All | Intraspecific richness | Insect richness | -0.02 | 59.41% |
| All | Intraspecific richness | Plant survival | 6.50 | 92.48% |
| All | Insect richness | Percent herbivory | 8.78 | 100.00% |
| All | Insect richness | Presence of damage | 6.63 | 96.73% |
| All | Insect richness | Variance in herbivory | 17.86 | 71.13% |
| All | Insect richness | Plant survival | 3.35 | 66.21% |
| All | Interspecific richness | Percent herbivory | 0.02 | 50.34% |
| All | Interspecific richness | Presence of damage | 7.40 | 83.01% |
| All | Interspecific richness | Variance in herbivory | -52.43 | 73.46% |
| All | Interspecific richness | Insect richness | 0.20 | 95.00% |
| All | Interspecific richness | Plant survival | 10.66 | 80.22% |
| All | Water availability | Percent herbivory | -4.19 | 98.73% |
| All | Water availability | Presence of damage | -6.33 | 89.46% |
| All | Water availability | Variance in herbivory | -118.10 | 91.35% |
| All | Water availability | Insect richness | -0.07 | 64.52% |
| All | Water availability | Plant survival | 5.15 | 79.29% |
| Costa Rica | Intraspecific richness | Percent herbivory | -0.10 | 52.68% |
| Costa Rica | Intraspecific richness | Presence of damage | 2.86 | 79.84% |
| Costa Rica | Intraspecific richness | Variance in herbivory | 37.95 | 72.01% |
| Costa Rica | Intraspecific richness | Insect richness | 0.10 | 98.07% |
| Costa Rica | Intraspecific richness | Plant survival | 3.72 | 80.91% |
| Costa Rica | Insect richness | Percent herbivory | 7.53 | 100.00% |
| Costa Rica | Insect richness | Presence of damage | 5.60 | 100.00% |
| Costa Rica | Insect richness | Variance in herbivory | -6.74 | 65.69% |
| Costa Rica | Insect richness | Plant survival | 3.60 | 66.25% |
| Costa Rica | Interspecific richness | Percent herbivory | -2.43 | 91.53% |
| Costa Rica | Interspecific richness | Presence of damage | -4.89 | 89.55% |
| Costa Rica | Interspecific richness | Variance in herbivory | -89.88 | 83.97% |
| Costa Rica | Interspecific richness | Insect richness | -0.08 | 93.43% |
| Costa Rica | Interspecific richness | Plant survival | 18.69 | 99.61% |
| Costa Rica | Water availability | Percent herbivory | -4.76 | 99.99% |
| Costa Rica | Water availability | Presence of damage | -7.96 | 99.90% |
| Costa Rica | Water availability | Variance in herbivory | -151.05 | 98.04% |
| Costa Rica | Water availability | Insect richness | -0.02 | 62.33% |
| Costa Rica | Water availability | Plant survival | 12.09 | 99.74% |
| Ecuador | Intraspecific richness | Percent herbivory | -3.50 | 99.87% |
| Ecuador | Intraspecific richness | Presence of damage | -8.10 | 99.95% |
| Ecuador | Intraspecific richness | Variance in herbivory | -80.24 | 92.45% |
| Ecuador | Intraspecific richness | Insect richness | -0.18 | 100.00% |
| Ecuador | Intraspecific richness | Plant survival | 20.46 | 100.00% |
| Ecuador | Insect richness | Percent herbivory | 8.68 | 100.00% |
| Ecuador | Insect richness | Presence of damage | 8.23 | 100.00% |
| Ecuador | Insect richness | Variance in herbivory | 22.77 | 97.99% |
| Ecuador | Insect richness | Plant survival | 7.46 | 81.29% |
| Ecuador | Interspecific richness | Percent herbivory | -1.74 | 89.56% |
| Ecuador | Interspecific richness | Presence of damage | 8.46 | 99.73% |
| Ecuador | Interspecific richness | Variance in herbivory | 16.01 | 57.91% |
| Ecuador | Interspecific richness | Insect richness | 0.58 | 100.00% |
| Ecuador | Interspecific richness | Plant survival | 7.78 | 85.86% |
| Ecuador | Water availability | Percent herbivory | -2.67 | 99.52% |
| Ecuador | Water availability | Presence of damage | -5.72 | 99.78% |
| Ecuador | Water availability | Variance in herbivory | -28.52 | 68.94% |
| Ecuador | Water availability | Insect richness | 0.04 | 85.57% |
| Ecuador | Water availability | Plant survival | 5.51 | 90.57% |
| Mogi | Intraspecific richness | Percent herbivory | -2.15 | 98.59% |
| Mogi | Intraspecific richness | Presence of damage | -4.29 | 97.74% |
| Mogi | Intraspecific richness | Variance in herbivory | -24.33 | 67.12% |
| Mogi | Intraspecific richness | Insect richness | 0.02 | 75.41% |
| Mogi | Intraspecific richness | Plant survival | 1.57 | 66.65% |
| Mogi | Insect richness | Percent herbivory | 11.17 | 100.00% |
| Mogi | Insect richness | Presence of damage | 12.22 | 100.00% |
| Mogi | Insect richness | Variance in herbivory | 80.94 | 99.99% |
| Mogi | Insect richness | Plant survival | 1.95 | 58.03% |
| Mogi | Interspecific richness | Percent herbivory | 2.02 | 90.42% |
| Mogi | Interspecific richness | Presence of damage | 6.39 | 97.05% |
| Mogi | Interspecific richness | Variance in herbivory | -116.52 | 90.64% |
| Mogi | Interspecific richness | Insect richness | 0.04 | 80.94% |
| Mogi | Interspecific richness | Plant survival | 17.14 | 99.05% |
| Peru | Intraspecific richness | Percent herbivory | -0.09 | 51.56% |
| Peru | Intraspecific richness | Presence of damage | -9.11 | 94.39% |
| Peru | Intraspecific richness | Variance in herbivory | -58.63 | 73.32% |
| Peru | Intraspecific richness | Insect richness | -0.03 | 63.89% |
| Peru | Intraspecific richness | Plant survival | 2.84 | 74.08% |
| Peru | Insect richness | Percent herbivory | 8.09 | 100.00% |
| Peru | Insect richness | Presence of damage | 4.38 | 99.51% |
| Peru | Insect richness | Variance in herbivory | 30.07 | 87.18% |
| Peru | Insect richness | Plant survival | -2.31 | 61.10% |
| Peru | Interspecific richness | Percent herbivory | 0.14 | 50.60% |
| Peru | Interspecific richness | Presence of damage | 16.49 | 97.23% |
| Peru | Interspecific richness | Variance in herbivory | -69.46 | 70.67% |
| Peru | Interspecific richness | Insect richness | 0.16 | 91.04% |
| Peru | Interspecific richness | Plant survival | 5.70 | 77.33% |
| Peru | Water availability | Percent herbivory | -5.15 | 99.75% |
| Peru | Water availability | Presence of damage | -5.27 | 89.79% |
| Peru | Water availability | Variance in herbivory | -173.87 | 94.77% |
| Peru | Water availability | Insect richness | -0.22 | 99.69% |
| Peru | Water availability | Plant survival | -2.10 | 69.02% |
| Uaimii | Intraspecific richness | Percent herbivory | 1.51 | 71.49% |
| Uaimii | Intraspecific richness | Presence of damage | 2.21 | 64.77% |
| Uaimii | Intraspecific richness | Variance in herbivory | 74.93 | 79.39% |
| Uaimii | Intraspecific richness | Insect richness | -0.01 | 55.41% |
| Uaimii | Intraspecific richness | Plant survival | 3.92 | 77.51% |
| Uaimii | Insect richness | Percent herbivory | 8.41 | 100.00% |
| Uaimii | Insect richness | Presence of damage | 2.83 | 85.39% |
| Uaimii | Insect richness | Variance in herbivory | -36.56 | 83.52% |
| Uaimii | Insect richness | Plant survival | 5.98 | 75.15% |
| Uaimii | Interspecific richness | Percent herbivory | 2.23 | 70.86% |
| Uaimii | Interspecific richness | Presence of damage | 10.61 | 87.21% |
| Uaimii | Interspecific richness | Variance in herbivory | -3.20 | 52.59% |
| Uaimii | Interspecific richness | Insect richness | 0.27 | 98.14% |
| Uaimii | Interspecific richness | Plant survival | 3.53 | 62.35% |
