# Supplementary material for: Intra- and interspecific diversity in a tropical plant clade alter herbivory and ecosystem resilience
Source: eLife. 2024 Apr 25;12:RP86988. doi: 10.7554/eLife.86988 (PMC11045218; doi:10.7554/eLife.86988)
Supplement: Supplementary file 2. — *Climate data from La Selva Biological station are for the experimental period (2015–2018) and were provided by the Organization for Tropical Studies. Data from El Fundo Génova are based on nearby San Ramón (https://en.climate-data.org/south-america/peru/junin/san-ramon-28556/). Data from Ecuador were provided by Yanayacu Biological Station. Data for Mogi-Guaçu Biological Reserve are from January 2017 to December 2019 and are from the Centro Integrado de Informações Agrometeorológicas of São Paulo. Data for Uaimii State Forest are from the Plano de Manejo FLOE Uaimii, Instituto Estadual de Florestas of Minas Gerais [file elife-86988-supp2.docx]

**Supplementary file 2.** Study site characteristics and experimental details

|  | La Selva Biological Station | Yanayacu Biological Station | El Fundo Génova | Mogi-Guaçu Biological Reserve | Uaimii  State Forest |
| --- | --- | --- | --- | --- | --- |
| Location | N9.9400, W84.0433 | S0.6008, W77.8903 | S11.0948, W75.3516 | S32.2510, W47.1558 | S30.2957,  W43.5731 |
| Mean elevation (m a.s.l.) | 35 | 2124 | 1133 | 627 | 800 |
| Mean annual precipitation (mm) | 4495 | 2900 | 1767 | 1271 | 1600 |
| Mean annual T (°C) | 25 | 16 | 23 | 26 | 19 |
| Date planted | 2015-03-01 | 2015-07-01 | 2015-10-01 | 2017-02-01 | 2018-02-20 |
| Date harvested | 2017-02-01 | 2016-12-01 | 2017-07-01 | 2019-12-01 | 2019-12-20 |
| Number of species in high richness plots | 12 | 12 | 6 | 4 | 3 |
| Planted in pots / ground | Pots | Pots | Pots | Ground | Ground |
| Water addition treatment applied | Yes | Yes | Yes | No | No |
| Number of *Piper* planted | 360 | 360 | 360 | 432 | 144 |
| Number of *Piper* surviving till the end of the experiment | 200 | 259 | 56 | 182 | 123 |

*Climate data from La Selva Biological station are for the experimental period (2015-2018) and were provided by the Organization for Tropical Studies. Data from El Fundo Génova are based on nearby San Ramón (<https://en.climate-data.org/south-america/peru/junin/san-ramon-28556/>). Data from Ecuador were provided by Yanayacu Biological Station. Data for Mogi-Guaçu Biological Reserve are from January 2017-December 2019 and are from the Centro Integrado de Informações Agrometeorológicas of São Paulo. Data for Uaimii State Forest are from the Plano de Manejo FLOE Uaimii, Instituto Estadual de Florestas of Minas Gerais
