# Supplementary material for: Intra- and interspecific diversity in a tropical plant clade alter herbivory and ecosystem resilience
Source: eLife. 2024 Apr 25;12:RP86988. doi: 10.7554/eLife.86988 (PMC11045218; doi:10.7554/eLife.86988)
Supplement: Supplementary file 3. — * indicates morphospecies. [file elife-86988-supp3.docx]

**Supplementary file 3.** Species of *Piper* used at each study location

| Study location | | | | |
| --- | --- | --- | --- | --- |
| La Selva Biological Station | Yanayacu Biological Station | El Fundo Génova | Mogi-Guaçu Biological Reserve | Uaimii  State Forest |
| arboreum | grande* | arboreum | arboreum | lepturum |
| peltatum | peludo* | reticulatum | crassinervium | corcovadense |
| biolleyi | ecuadorense | glabribaccum | richardifolium | vicosanum |
| sancti-felicis | perareolatum | chanchamayanum | miquelianum |  |
| reticulatum | baezanum | armatum |  |  |
| nudifolium | escabrosa | lechlerianum |  |  |
| multiplinervium | kelleii |  |  |  |
| decurrens | hispidum |  |  |  |
| pseudobumbratum | schupii |  |  |  |
| imperiale | pubinervulum |  |  |  |
| garagaranum | pequenia* |  |  |  |
| urostachyum |  |  |  |  |

* Indicates morphospecies
